# Combined evaluation of the FAS cell surface death receptor and CD8+ tumor infiltrating lymphocytes as a prognostic biomarker in breast cancer

**DOI:** 10.18632/oncotarget.14779

**Published:** 2017-01-21

**Authors:** Erik J. Blok, Jitske van den Bulk, N. Geeske Dekker-Ensink, Remco Derr, Corné Kanters, Esther Bastiaannet, Judith R. Kroep, Cornelis J.H. van de Velde, Peter J.K. Kuppen

**Affiliations:** ^1^ Department of Surgery, Leiden University Medical Center, Leiden, The Netherlands; ^2^ Department of Medical Oncology, Leiden University Medical Center, Leiden, The Netherlands

**Keywords:** FAS, biomarker, tumor infiltrating lymphocytes, CD8, breast cancer

## Abstract

Multiple studies showed the prognostic capacities of tumor-infiltrating lymphocytes (TILs) in triple-negative breast cancer (TNBC), but not in other subtypes. We evaluated tumor expression of FAS, a key receptor in T-cell mediated apoptosis, as possible explanation for this differential prognostic value of TILs. Furthermore, we evaluated the prognostic relevance of FAS, both as an independent biomarker and in relation to CD8-positive T-cell presence. The study cohort consisted of 667 breast cancer patients treated in the LUMC between 1997 and 2009. FAS expression was determined using immunohistochemistry and the percentage of FAS-positive tumor cells was quantified. Furthermore, the number of CD8-positive infiltrating cells was determined, and its prognostic relevance was associated to FAS-expression using stratified survival analysis. In TNBC, FAS was averagely expressed in 49% of tumor cells, whereas ER-positive subtypes showed an average Fas expression of 16-20%. In the entire cohort, FAS was identified as significant prognostic marker for recurrence (adjusted HR 0.53, 95% CI 0.36-0.77) and borderline significant marker for overall survival (adjusted HR 0.72, 95% CI 0.52-1.01). Upon stratification for FAS-expression, CD8+ TILs were only prognostic at high levels (above median) of FAS expression in ER-negative disease. In summary, FAS was identified as an independent prognostic marker for recurrence free survival in breast cancer, with large variation in expression by receptor subtypes. Interestingly, the prognostic effect of CD8+ TILs in ER-negative disease was only valid for tumors with a high FAS expression.

## INTRODUCTION

For decades the local immune response, among others represented by quantification of tumor-infiltrating lymphocytes (TILs), has been studied in breast cancer [1–3]. Although most studies observed a prognostic value of TILs, these studies have not resulted in any change in clinical practice. Studies have shown TILs to have strong prognostic impact in ER-negative, HER2-negative and triple negative breast cancer (TNBC), but not in ER-positive tumors [4–14]. In a recent meta-analysis by Ibrahim *et al* combining the results of 8 studies, a 30% reduction in disease recurrences and a 22% decrease in distant recurrences was shown for triple-negative patients having high amounts of TILs [14]. Furthermore, a hazard ratio 0.66 (95% CI 0.53-0.83) for overall survival was shown for these patients, providing robust evidence for the prognostic value of TILs.

It is known that although TILs might be present in the tumor, their functioning might be hampered [3]. One of the most studied factors involved is classical HLA class I, which was shown to be downregulated in breast cancer and other malignancies [15, 16]. Another protein on tumor cells that determines function of T cells is Fas cell surface death receptor, abbreviated as FAS. FAS is broadly expressed on most normal tissue, and is a crucial link between T-cell mediated immunity and induction of apoptosis [17, 18]. When a cytotoxic T-cell binds to a target cell, FAS-ligand (FASL) is upregulated by the T-cell. FASL subsequently binds to the target cell-expressed FAS, thereby initiating the activation of a caspase cascade leading to apoptosis of the target cell. Together with perforin-induced apoptosis, these are the two main mechanisms by which a cytotoxic T-cell can induce apoptosis [19, 20]. It could be hypothesized that downregulation of FAS is a mechanism of tumor immune evasion, since this disables a crucial step in T-cell mediated immunity. Therefore, tumor expression of FAS could act as a clinical prognostic marker in breast cancer.

Hypothetically, the expression of FASL by tumor cells could lead to induction of apoptosis in the cytotoxic T-cells which could be a second method of FAS-FASL-mediated immune evasion. A number of studies have been performed evaluating the prognostic relevance of FAS and FASL in breast cancer, focusing mainly on the FASL/FAS ratio [21–23]. These studies indeed reported that a higher tumor expression of FASL and/or a lower expression of FAS, resulting in an increased FASL/FAS ratio, associated with a worse disease free and overall survival [21]. Other studies reported that this was mainly due to an increase in FAS-expression, whereas FASL did not influence outcome [23]. Furthermore, the theory of immune evasion by upregulation of FASL in the tumor has never been shown *in vivo* [24]. Therefore, it is expected that most effects seen for the FASL/FAS ratio in tumors are attributed to a downregulation of FAS.

Although TILs have shown to be of prognostic relevance, it is highly unlikely that the TILs in the primary tumor will determine survival outcome. Most likely the amount of TILs in the primary tumor is a proxy variable for a yet undefined tumor characteristic, making the tumor more or less susceptible for an immune response. This process could lead to an aberrant pattern of metastasizing, or an effect on growth speed of the metastasis. When FAS is differentially expressed among different tumor subtypes, it could be hypothesized that FAS is a key explanatory factor for the fact that TILs are prognostic in one subgroup, but not in other subgroups. Furthermore, combining recent evidence regarding TILs in TNBC with the earlier evidence on FAS expression, we suggest that FAS is a clinical prognostic in breast cancer as an independent alternative for TILs.

Therefore, three main aims of this study are identified: To evaluate the expression of FAS among different tumor subtypes in order to explain variances in the prognostic value of TILs. The second aim is to evaluate the expression of FAS as a prognostic marker in breast cancer, both in general and in selected subtypes. Finally, the third aim of this study was to evaluate the prognostic value of CD8 in the presence or absence of FAS-expression, since we hypothesize that CD8-positive T-cells will only be prognostic in the presence of tumor FAS expression

## RESULTS

### Baseline characteristics

667 patients were included in this observational cohort of patients treated in the LUMC (Table 1). Most tumors were categorized as ductal carcinomas (80,8%); 10,2% were determined to be lobular carcinoma. Approximately 75% of the tumors showed ER positivity, 55% PR positivity and 25% HER2 positivity. For HER2 expression, nearly 50% of the records was missing. Missing of these data was strongly correlated to the year of diagnosis. Before 2003, 89% of HER2 scores was missing (322 of 360 patients), whereas from 2003 onwards it was only missing in 5% of patients (14 of 307 patients). The percentage of triple negative tumors was 16%, whereas ER+PR+HER2- was the most prevalent subtype with 42%. The majority of tumors were small and early stage (stage II or lower), only approximately 10% was stage III or IV. Most patients (91,8%) did not receive neoadjuvant systemic therapy. These percentages show that the cohort is representative for the general breast cancer population.

**Table 1:** Baseline overview of the clinicopathological parameters of the cohort

		Cohort description		Fas expression by medianN=640 (27 excluded)				Fas expression	CD8+ TILs by medianN=640 (42 excluded)				CD8+ TILs
		Total N=667		Low(< median)		High(> median)		Mean (%)	Low(< median)		High(> median)		Mean (n)
		N	%	N	%	N	%		N	%	N	%	
Age	<40	55	8,2%	17	30,9%	38	69,1%*	34	13	23,6%	42	76,4%*	69
	40-49	153	22,9%	67	46,2%	78	53,8%	23	55	37,7%	91	62,3%*	54
	50-59	210	31,5%	96	47,3%	107	52,7%	22	109	53,7%	94	46,3%	43
	60-69	127	19,0%	70	56,5%*	54	43,5%	19	74	60,7%*	48	39,3%	35
	>70	122	18,3%	56	49,6%	57	50,4%	21	70	61,4%*	44	38,6%	37
Histological subtype	ductal	539	80,8%	248	48,1%	268	51,9%	23	252	49,1%	261	50,9%	47
	lobular	68	10,2%	32	47,8%	35	52,2%	18	37	54,4%	31	45,6%	36
	other	50	9,0%	26	45,6%	31	54,4%	23	32	54,2%	27	45,8%	42
Bloom & Richardson grade	grade 1	108	18,2%	47	46,5%	54	53,5%	20	57	57,0%	43	43,0%	38
	grade 2	275	46,5%	136	52,3%	124	47,7%	19	146	55,9%	115	44,1%	36
	grade 3	209	35,3%	89	43,4%	116	56,6%	29	79	38,7%	125	61,3%*	62
	missing	75	-	-	-	-	-	-	-	-	-	-	-
ER expression (>10%)	no	140	23,5%	48	35,3%	88	64,7%*	37	55	40,4%	81	59,6%*	64
	yes	456	76,5%	218	49,9%	219	50,1%	19	230	53,0%	204	47,0%	39
	missing	71	-	-	-	-	-	-	-	-	-	-	-
PgR expression (>10%)	no	261	45,2%	104	41,4%	147	58,6%*	29	116	45,8%	137	54,2%	55
	yes	316	54,8%	153	50,5%	150	49,5%	19	160	53,5%	139	46,5%	37
	missing	90	-	-	-	-	-	-	-	-	-	-	-
HER2 expression	no	247	74,6%	106	44,4%	133	55,6%	23	131	54,4%	110	45,6%	43
	yes	84	25,4%	45	55,6%	36	44,4%	21	36	44,4%	45	55,6%	50
	missing	336	-	-	-	-	-	-	-	-	-	-	-
Receptor subtype	ER-PR-HER2-	52	16,0%	8	17,4%	38	82,6%*	49	18	36,7%	31	63,3%	79
	ER-PR-HER2+	31	9,5%	15	48,4%	16	51,6%	29	11	35,5%	20	64,5%	54
	ER+PR-HER2-	47	14,4%	25	53,2%	22	46,8%	16	29	61,7%	18	38,3%	32
	ER+PR-HER2+	25	7,7%	13	52,0%	12	48,0%	16	12	48,0%	13	52,0%	45
	ER-PR+HER2-	9	2,8%	4	57,1%	3	42,9%	20	5	71,4%	2	28,6%	27
	ER+PR+HER2-	137	42,0%	68	49,6%	69	50,4%	18	78	57,4%	58	42,6%	35
	ER+PR+HER2+	25	7,7%	17	68%*	8	32,0%	16	12	50,0%	12	50,0%	52
	missing	341	-	-	-	-	-	-	-	-	-	-	-
Tumor stage based on pT, pN and p/cM	IA	248	39,3%	109	46,4%	126	53,6%	21	127	53,6%	110	46,4%	38
	IB	4	0,6%	3	75,0%	1	25,0%	9	1	25,0%	3	75,0%	40
	IIA	184	29,2%	84	46,7%	96	53,3%	25	79	44,9%	97	55,1%	56
	IIB	133	21,0%	60	48,4%	64	51,6%	22	61	48,0%	66	52,0%	45
	IIIA	23	3,6%	11	50,0%	11	50,0%	27	9	40,9%	13	59,1%	55
	IIIB	5	0,8%	4	80,0%	1	20,0%	4	3	60,0%	2	40,0%	43
	IIIC	30	4,7%	15	50,0%	15	50,0%	21	17	56,7%	13	43,3%	43
	IV	4	0,6%	4	100,0%	0	0,0%	5	2	50,0%	2	50,0%	62
	missing	35	-	-	-	-	-	-	-	-	-	-	-
Neoadjuvant systemic therapy	CT	32	4,8%	12	40,0%	18	60,0%	26	18	60,0%	12	40,0%	34
	HT	22	3,3%	12	54,5%	10	45,5%	19	10	45,5%	12	54,5%	39
	CT + HT	1	0,1%	1	100,0%	0	0,0%	3	0	0,0%	1	100,0%	102
	none	612	91,8%	281	47,9%	306	52,1%	23	293	49,9%	294	50,1%	46

Both FAS-expression and presence of CD8+ tumor infiltrating lymphocytes (TILs) are shown, stratified according to standard clinicopathological parameters. Percentages are excluding missing variables. *column proportion test p-value <0.05

### FAS-expression

**Figure 1:**
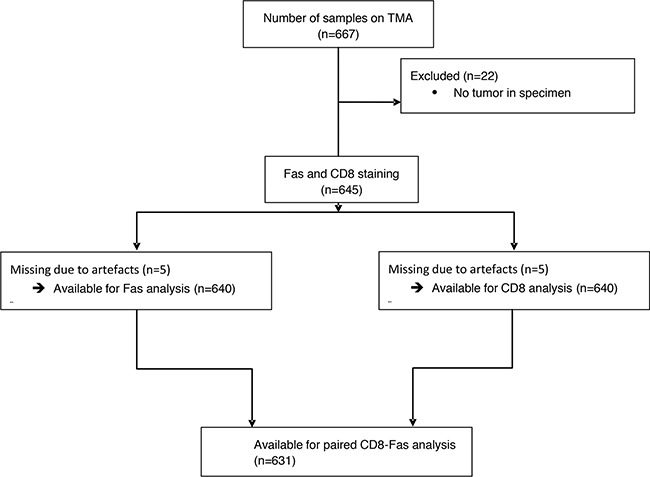
Consort diagram of the included patients, which were present on the TMA, for analysis The causes for missing samples were a lack of tumor in the punches or artefacts like folded or missing parts of the punches.

**Figure 2:**
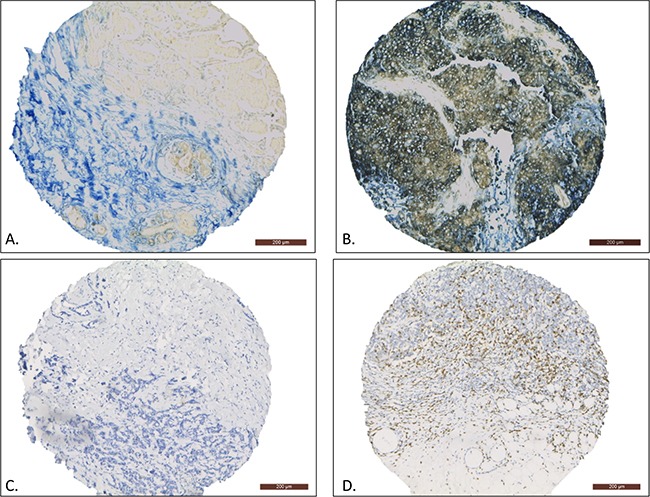
Representative examples of immunohistochemical staining for FAS expression and CD8-positive TILs (10x magnification) The FAS-negative sample only contains some FAS-positive infiltrating lymphocytes **A**. whereas the FAS-positive sample shows homogenous membranous FAS expression in the tumor cells **B**. The CD8-low sample showed no infiltration of CD8-positive TILs **C**. whereas the CD8-high sample shows large numbers of CD8-positive TILs **D**.

**Figure 3:**
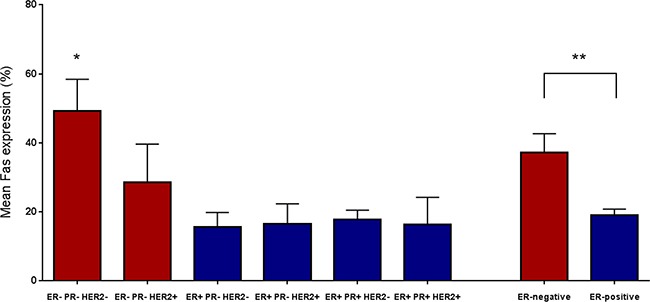
The average percentage of FAS expressing tumor cells, as determined by immunohistochemical staining, according to molecular subtypes *Significantly different from all other individual subgroups using Bonferroni's multiple comparisons test (all adjusted p-values <0.001). ** Unpaired t-test p-value <0.001.

From the 667 patients included in this observational cohort, immunohistochemical staining for FAS expression was successful for 640 patients. 27 patients were excluded due to a lack of tumor tissue on the TMA, either as an artefact or because only non-tumorous tissue was included on the TMA (Figure 1). In the remaining 640 patients, FAS expression was observed ranging from 0% to 100% of the tumor cells, with a median expression of 13.3% (Figure 2). The correlations with baseline characteristics are shown in Table 1. A small difference in FAS expression was shown for age, in which younger (<40 years) patients showed a higher expression of FAS, whereas patients between 60 and 69 showed a slightly lower FAS-expression (column proportion test p-value <0.05). No associations were found for histological subtype or tumor stage. It was observed that grade 3 tumors had a significantly higher FAS expression compared to grade 1 and 2. ER-negative tumors showed almost a doubling of the average expression of FAS compared to ER-positive tumors (37% vs 19% FAS-positive tumor cells per sample, p<0.05)). For HER2, limited data were available (n=320), showing no statistical differences. Combining ER, PR and HER2, it was shown that triple negative tumors showed significantly higher FAS-expression (average of 49% positive tumor cells) compared to the other subtypes (Bonferroni multiple comparisons test p-values <0.001), especially ER-positive subtypes (FAS expression ranging from 16% to 18% positive tumor cells) (Figure 3). Pre-treatment with either neo-adjuvant chemotherapy or endocrine therapy was not associated with different FAS-expression patterns.

In the provisional TCGA dataset, levels of FAS expression were compared to the expression of ESR1, the gene encoding for ER. We observed a Pearson correlation of -0.35, meaning that a higher FAS expression is correlated to a lower expression of ER. This is in accordance with our findings that in ER-negative tumors, there is a higher FAS expression. To supplement these findings, we analyzed the TCGA dataset as published in Nature in 2012, for which more clinical data are available [25]. In this cohort, we observed that of the 14 patients who have an upregulation of FAS at transcriptional mRNA level, 13 of them are ER-negative and for one patient ER-staining was not performed. In contrast, of the 17 patients with a downregulation of FAS, 13 were ER-positive. This further validated our finding, that high levels of FAS are associated to low levels of ER-expression, both at transcriptional and protein level.

### FAS-expression as clinical prognostic marker

**Figure 4:**
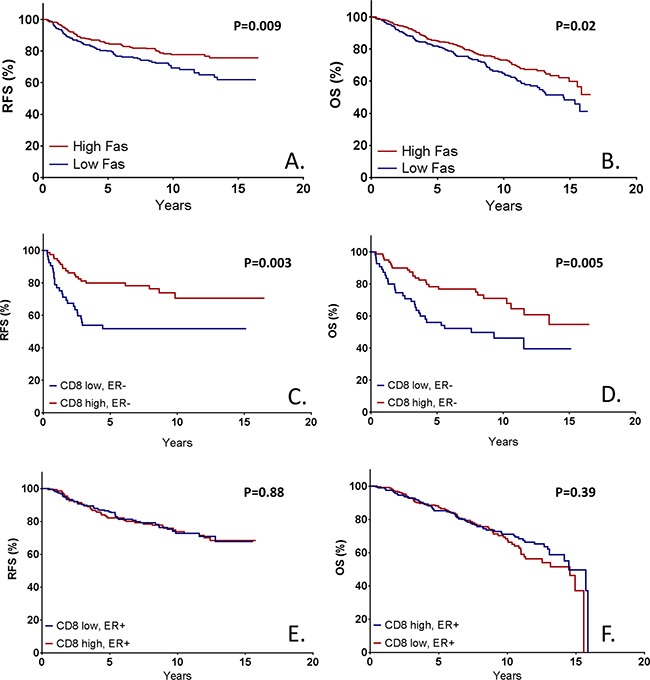
Kaplan Meier survival estimates based on immunohistochemical FAS expression both for recurrence free survival **A**. and overall survival **B**. Furthermore, the prognostic effect of CD8-positive TILs is shown in both ER-negative (**C**. RFS, **D**. OS) and ER-positive tumors (**E**. RFS, **F**. OS). P-values represent log-rank survival test.

To evaluate the clinical prognostic value of FAS-expression, Kaplan Meier curves were plotted for the general study population (Figure 4A, 4B). It was shown that a high FAS expression correlated with a longer recurrence free and overall survival time (log-rank p-values of 0.009 and 0.02 respectively) in the entire cohort. In a univariate cox-regression analysis, a hazard ratio of 0.65 (95% CI 0.47-0.90, p=0.01) and 0.72 (95% CI 0.55-0.95, p=0.02) was seen for RFS and OS respectively. In a multivariate cox regression analysis, corrected for age, histological subtype, tumor grade, tumor stage, ER-expression, year of diagnosis, neo-adjuvant treatment, adjuvant chemotherapy, and adjuvant endocrine therapy, an adjusted HR of 0.53 (95% CI 0.36-0.77, p=0.001) was seen for RFS. For OS, an adjusted HR of 0.72 was observed, with a borderline significance (95% CI 0.52-1.01, p=0.055).

Upon stratification on ER-expression, it was shown that both in ER-negative (HR 0.48, 95% CI 0.27-0.86, p=0.01) and ER-positive (HR 0.65, 95% CI 0.43-0.97, p=0.04) tumors, FAS expression was prognostic for RFS (Supplementary Figure 2A,B). In multivariate analysis, a HR of 0.36 (95% CI 0.17-0.76, p=0.01) was shown in ER-negative tumors, whereas a HR of 0.58 (95% CI 0.37-0.90, p=0.02) was shown for ER-positive tumors. For OS, no statistical significant differences regarding level of FAS expression were shown for ER-negative tumors in univariate (HR 0.78, 95% CI 0.45-1.35, p=0.38) or multivariate (HR 0.65, 95% CI 0.33-1.30, p=0.23) modelling (Supplementary Figure 2C). In ER-positive tumors, a strong benefit of FAS-expression was shown for OS (HR 0.59, 95% CI0.42-0.83, p=0.003), but this failed to show in multivariate analysis (HR 0.76, 95% CI 0.52-1.12, p=0.16) (Supplementary Figure 2D). No significant interaction between ER-status and FAS-expression was observed for either RFS or OS (HRs 0.70 (p=0.32) and 1.15 (p=0.66) respectively), meaning that the effect of FAS expression on survival is not significantly different between ER-negative and ER-positive patients.

In summary, an above median level of FAS expression was a statistically significant independent prognostic marker for RFS, and a borderline significant prognostic marker for OS. Both effects were conserved in ER-negative and ER-positive tumors.

### CD8+ lymphocyte infiltration

For the evaluation of CD8+ tumor infiltrating lymphocytes (TILs), TILs were counted in both the tumor and the directly adjacent stromal tissue, only when the punch contained tumor tissue. Therefore, 27 cases were excluded due to artefacts (missing punches) or a lack of tumor tissue in the sample. For the remaining 640 patients, the number of CD8+ TILs ranged from 0 to 369, with a median value of 28 per punch (1mm). The distribution of CD8+ TIL infiltration over basic clinicopathological subgroups is shown in Table 1. Young patients (<40 and 40-49) showed an increased amount of infiltration compared to other age categories. Furthermore, ER-negative and grade 3 tumors showed increased rates of infiltration.

Over the whole cohort, CD8+ TILs showed no correlation with RFS (HR 1.16, 95% CI 0.84-1.60, p=0.36) (Supplementary Figure 1A). However, when corrected for age, histological subtype, tumor grade, tumor stage, ER-expression, year of diagnosis, neo-adjuvant treatment, adjuvant chemotherapy, and adjuvant endocrine therapy, an adjusted HR of 0.55 (95% CI 0.37-0.81, p=0.003) was observed for RFS for patients with an above-median level of CD8+ TILs. Similar effects were shown for OS (HR 1.23, 95% CI 0.94-1.62, p=0.14; adjusted HR 0.78, 95% CI 0.56-1.10, p=0.16), although not statistically significant (Supplementary Figure 1B).

In earlier studies, it was observed that CD8+ TILs were only prognostic in ER-negative or triple negative breast cancer [4]. Upon stratification on ER-expression, similar results were observed in this cohort (Figure 4C–4F). In ER-positive patients, high levels of CD8+ TILs were associated with a HR for recurrence of 1.03 (95% CI 0.69-1.54, p=0.88), whereas in ER-negative disease a HR of 0.42 (95% CI 0.23-0.76, p=0.004) was observed (HR for interaction 2.64, p=0.007). A similar pattern was observed for OS (ER+ HR 0.86, 95% CI 0.61-1.21, p=0.39; ER- HR 0.48, 95% CI 0.28-0.81, p=0.007).

### Combined FAS-CD8 analysis

In order to determine the hypothesized pivotal effect of FAS expression on the function, and therefore prognostic effect of CD8+ TILs, a survival analysis was performed on the presence of CD8+ lymphocytes, stratified on FAS expression. In the complete cohort, there was no difference between the prognostic relevance of CD8 TILs for either high or low expression of FAS on RFS (HR for interaction 1.51, p=0.24) or OS (HR for interaction 1.24, p=0.45).

**Figure 5:**
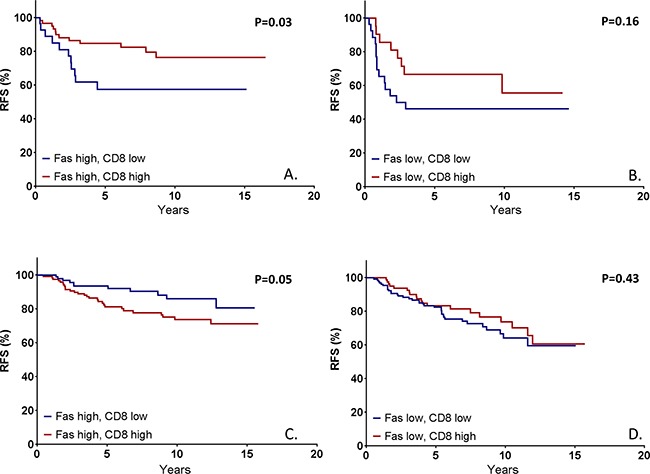
Kaplan Meier survival estimates based on CD8+ tumor infiltrating lymphocytes (TILs) stratified on FAS expression in both ER-negative and ER-positive tumors In patients with ER-negative tumors with high FAS expression, the high presence of CD8-positive is prognostic for a significantly higher survival **A**. This prognostic effect is not observed in ER-negative tumors with low FAS expression **B**. nor in ER-positive tumors **C, D**. P-values represent log-rank survival test.

Upon stratification on ER expression, a similar analysis was performed. In ER-negative disease, it was shown that CD8 was prognostic for RFS in the presence of high FAS expression (HR 0.42, 95% CI 0.19-0.96, p=0.04), but not with low FAS expression (HR 0.54, 95% CI 0.23-1.29, p=0.17), with a HR for interaction of 0.80, (p=0.71) (Figure 5A,B). In ER-positive disease, CD8 was reversely prognostic for RFS with at high levels of FAS expression (HR 2.01, 95% CI 1.01-4.04, p=0.05) and not prognostic at low FAS expression (HR 0.80, 95% CI0.46-1.39, p=0.43), with a HR for interaction of 2.42, p=0.05 (Figure 5C,D). For OS, a similar pattern was observed (ER-negative, high FAS: HR 0.49 (95% CI 0.25-0.97, p=0.04), ER-negative, low FAS: HR 0.51 (95% CI 0.20-1.26, p=0.14) (Supplementary Figure 3A,B); ER-positive, high FAS: HR 1.22 (95% CI 0.70-2.12, p=0.48), ER-positive, low FAS: HR 0.78 (95% CI 0.48-1.25, p=0.30) (Supplementary Figure 3C,D). In summary, CD8+ TILs were only prognostic for both RFS and OS in ER-negative tumors at high levels of FAS, but not in tumors with low expression of FAS or in ER+ tumors.

## DISCUSSION

This study is the first study to determine the influence of the immune-editing protein FAS on the prognostic value of TILs. Our studies shows that besides ER-negative status, also a positive FAS-status is required for CD8+ TILs to be prognostic in breast cancer.

Furthermore, we assess the value of FAS as an independent prognostic marker. It was shown that patients with a higher FAS-expression have a longer recurrence free and overall survival, even when corrected for age, histological subtype, tumor grade, tumor stage, ER-status, year of diagnosis, neo-adjuvant treatment, adjuvant chemotherapy and adjuvant endocrine therapy. This indicates that, irrespective of the amount of TILs, FAS serves as a prognostic marker. In our subgroup analysis, we confirmed that this effect was conserved in both ER-negative and ER-positive disease for recurrence free survival, but not for overall survival.

We also observed that FAS was expressed in nearly twice as much tumor cells in ER-negative tumors, compared to ER-positive tumors, which also explains the higher expression of FAS in younger patients, since those are more often ER-negative [26]. Even more, triple negative tumors had more than twice as much FAS-positive tumor cells compared to ER-positive tumors. This difference in FAS expression might explain the observation in earlier studies that infiltrating lymphocytes are only prognostic in ER-negative or triple negative tumors [4]. Since T-cell mediated immunity depends on FAS-expression of the target cell, a higher expression of FAS as observed in TNBC may render the cells more susceptible for infiltrating T-cells. With lower amounts of FAS-expression, as observed in ER-positive disease, infiltrating T-cells may have less possibilities to induce apoptosis, and will therefore be much less or even not prognostic since their functioning will be hampered. This explains why in ER-positive disease there is no additional value of CD8+ TILs over the prognostic value of FAS. However, we observed that in ER-positive tumors with high expression of FAS, there was even a significant negative effect of CD8-positive TILs on survival. This indicates that there is a FAS-independent, unknown factor which prevents CD8-positive TILs from functioning in ER-positive breast cancer Furthermore, since FAS itself is prognostic in ER-positive breast cancer, it indicates that there are more anti-tumor mechanisms of FAS expression besides the T-cell mediated immunity, which were described in detail earlier [27]. These mechanisms could have contributed to the favorable prognostic effect of FAS expression on clinical survival.

In ER-negative disease, it was observed that CD8+ TILs were only prognostic in the presence of high FAS expression, confirming the pivotal role of FAS in T-cell mediated immunity. Recently, immunotherapy has gained many interest in different fields of oncology, including breast cancer [28, 29]. These therapies are based on targeted agents (e.g. PD1 or PDL1 inhibitors), which enhance the immune response against the tumor [30]. We observed that TILs are only prognostic, and therefore perhaps are only functional, in the presence of FAS expression in ER-negative breast cancer. It could be hypothesized that FAS expression might therefore act as a predictive factor for these new emerging therapies.

Due to the retrospective, observational design of the cohort, there are some limitations to this study. First, HER2 was missing in nearly half of all patients based on the year of diagnosis, limiting the power for HER2-specific subgroup analyses). Therefore the year of diagnosis could have acted as a confounder, influencing both HER2-expression and outcome. To overcome this confounding, the year of diagnosis was included as a corrective factor in multivariate analysis. Furthermore, since including HER2 in the multivariate model would lead to a skewed cohort only consisting of patients diagnosed after 2003, HER2 was not included as a corrective factor in multivariate analysis.

In summary, this study is the first study reporting a differential expression of FAS among tumors with different receptor subtypes; TNBC shows nearly twice as much expression compared to other subtypes. Furthermore, we showed that FAS is an independent prognostic marker in breast cancer, independent from estrogen receptor status or other possibly confounding factors. Finally, we showed that in ER-negative disease, FAS expression is necessary for the prognostic effect of TILs.

## MATERIALS AND METHODS

### Patient cohort

The cohort of patients used for this study consisted of a consecutive series of female breast cancer patients treated in the Leiden University Medical Center (LUMC) with surgery between 1997 and 2009 (n=667). Data regarding age, year of diagnosis, estrogen and progesterone receptor expression, human epidermal growth factor receptor 2 (HER2) expression (when available), TNM stage [31], tumor differentiation grade [32] and morphology, local and systemic therapy, secondary tumors, local, regional, distant, recurrence free and overall survival time and status was known for these patients. Formalin-fixed paraffin-embedded (FFPE) tumor samples were collected, and a tissue microarray (TMA) was created with three 1mm tumor tissue punches from each tumor.

### Immunohistochemistry

For immunohistochemical staining of FAS, 4.5 μm slides were cut from the aforementioned TMA and stored at +4 °C until use. Colon tissue was shown to be positive for FAS expression, therefore this tissue was used as positive control [18]. Slides were deparaffinized in xylene and rehydrated in serial dilutions of ethanol-H_2_O. Antigen retrieval was performed by placing the section at 95°C for 10 minutes in Target Retrieval Buffer Low pH (DAKO) in a PT Link (DAKO). Endogenous peroxidase and phosphatase was blocked by incubation of the sections in BloxAll (Vector, Burlingame, USA) for 10 min. LS-B2820 (LifeSpan BioSciences, Seattle, USA) was used as anti-FAS antibodies for IHC. The antibodies were diluted in phosphate-buffered saline with 1% of bovine serum albumin (1% PBS/BSA), and the optimal dilution was determined by titration. Incubations with primary antibodies were performed overnight. Envision HRP-labeled polymer anti-mouse (Dako, Carpinteria, USA) was used as secondary antibodies and incubated for 30 minutes. Slides were developed with DAB (Dako, Carpinteria, USA). Similar procedures were performed for a staining against CD8 to identify CD8+ cytotoxic T-cells (clone 4B11, Monosan).

In order to allow specific scoring of epithelial tumor cells, a counterstaining against stroma was performed using anti-rabbit polyclonal antibodies ab34710, ab6588 and ab23747 (Abcam, Cambridge, UK), targeting collagen I and IV and elastin respectively. Swine-anti-rabbit-AP (Dako, Carpinteria, USA) was used as secondary antibodies, incubated for 30 minutes and developed in the dark using VectorBlue Kit (Vector, Burlingame, USA). Finally, methyl green (Vector, Burlingame, USA) was used for staining of the nuclei. For this purpose, the section were incubated with methyl green for 5 min at 56 °C. After washing with demineralized water followed by acetone-HAc 0.05%, the sections were dehydrated by gradients of ethanol and dried by dipping in xylene. Slides were mounted in Vectormount (Vector, Burlingame, USA) and stored until further analysis.

### Quantification of IHC stainings

The Philips Ultra Fast Scanner 1.6 RA (Philips, Eindhoven, the Netherlands) was used for digitalization of the immunohistochemically stained sections of the TMA. For FAS, the percentage of tumor cells showing membranous staining was assessed by two independent observers. The scores of the three punches were combined to one average score per patient. Based on the whole cohort, the median value was used as a cut-off value to create a dichotomous value distinguishing low and high expression of FAS. For the evaluation of CD8, the number of CD8+ cells in the tumor was counted per punch, and the average of three punches was used for dichotomization based on the median value. Punches were only analyzed when more than 30% consisted of tumor tissue. Images were acquired using a Leica ICC50 camera system (Leica Microsystems, Wetzlar, Germany).

### mRNA expression analysis

To assess the correlation between FAS expression and ER-status at transcriptional level, the publicly available TCGA dataset was used using cBioPortal to assess the levels of mRNA gene expression, in comparison to clinical ER-status and the expression of ESR1, the gene encoding for ER [33, 34].

### Statistical analysis

SPSS (version 23 for Windows) was used for statistical analysis. Chi-square, column proportion tests, Bonferroni's multiple comparisons test and unpaired t-tests were used to identify associations between FAS expression, CD8+ TIL presence and baseline clinicopathological parameters. Kaplan Meier analysis was used to calculate recurrence free (RFS) and overall survival (OS) for the complete cohort and subgroups; log-rank tests were used to assess any differences between survival curves. RFS was defined as the time without local, regional or distant recurrence, whereas OS was defined as death from any cause. Death from breast cancer (disease-specific survival) was not recorded for this cohort. Cox regression analysis was used for univariate and multivariate analyses for RFS and OS. Furthermore, interaction tests were performed, to assess the marker interaction effect. This test assesses whether the prognostic value of a marker in one subgroup is significantly different from its value in a different subgroup. For all tests, p-values <0,05 were considered to be significant.

## SUPPLEMENTARY MATERIALS FIGURES AND TABLES



## References

[1] Denkert C, Loibl S, Noske A, Roller M, Müller BM, Komor M, Budczies J, Darb-Esfahani S, Kronenwett R, Hanusch C, von Törne C, Weichert W, Engels K (2010). Tumor-Associated Lymphocytes As an Independent Predictor of Response to Neoadjuvant Chemotherapy in Breast Cancer. Journal of Clinical Oncology.

[2] Whitford P, Mallon EA, George WD, Campbell AM (1990). Flow cytometric analysis of tumour infiltrating lymphocytes in breast cancer. Br J Cancer.

[3] Marrogi AJ, Munshi A, Merogi AJ, Ohadike Y, El-Habashi A, Marrogi OL, Freeman SM (1997). Study of tumor infiltrating lymphocytes and transforming growth factor-b as prognostic factors in breast carcinoma. Int J Cancer.

[4] Mahmoud SMA, Paish EC, Powe DG, Macmillan RD, Grainge MJ, Lee AHS, Ellis IO, Green AR (2011). Tumor-Infiltrating CD8+ Lymphocytes Predict Clinical Outcome in Breast Cancer. Journal of Clinical Oncology.

[5] West NR, Kost SE, Martin SD, Milne K, deLeeuw RJ, Nelson BH, Watson PH (2013). Tumour-infiltrating FOXP3(+) lymphocytes are associated with cytotoxic immune responses and good clinical outcome in oestrogen receptor-negative breast cancer. Br J Cancer.

[6] Liu S, Lachapelle J, Leung S, Gao D, Foulkes WD, Nielsen TO (2012). CD8(+) lymphocyte infiltration is an independent favorable prognostic indicator in basal-like breast cancer. Breast Cancer Res.

[7] West NR, Milne K, Truong PT, Macpherson N, Nelson BH, Watson PH (2011). Tumor-infiltrating lymphocytes predict response to anthracycline-based chemotherapy in estrogen receptor-negative breast cancer. Breast Cancer Res.

[8] Ali HR, Provenzano E, Dawson SJ, Blows FM, Liu B, Shah M, Earl HM, Poole CJ, Hiller L, Dunn JA, Bowden SJ, Twelves C, Bartlett JMS (2014). Association between CD8+ T-cell infiltration and breast cancer survival in 12 439 patients. Annals of Oncology.

[9] Baker K, Lachapelle J, Zlobec I, Bismar TA, Terracciano L, Foulkes WD (2011). Prognostic significance of CD8+ T lymphocytes in breast cancer depends upon both oestrogen receptor status and histological grade. Histopathology.

[10] Adams S, Gray RJ, Demaria S, Goldstein L, Perez EA, Shulman LN, Martino S, Wang M, Jones VE, Saphner TJ, Wolff AC, Wood WC, Davidson NE (2014). Prognostic Value of Tumor-Infiltrating Lymphocytes in Triple-Negative Breast Cancers From Two Phase III Randomized Adjuvant Breast Cancer Trials: ECOG 2197 and ECOG 1199. Journal of Clinical Oncology.

[11] Loi S, Michiels S, Salgado R, Sirtaine N, Jose V, Fumagalli D, Kellokumpu-Lehtinen PL, Bono P, Kataja V, Desmedt C, Piccart MJ, Loibl S, Denkert C (2014). Tumor infiltrating lymphocytes are prognostic in triple negative breast cancer and predictive for trastuzumab benefit in early breast cancer: results from the FinHER trial. Annals of Oncology.

[12] Loi S, Sirtaine N, Piette F, Salgado R, Viale G, Van Eenoo F, Rouas G, Francis P, Crown JPA, Hitre E, de Azambuja E, Quinaux E, Di Leo A (2013). Prognostic and Predictive Value of Tumor-Infiltrating Lymphocytes in a Phase III Randomized Adjuvant Breast Cancer Trial in Node-Positive Breast Cancer Comparing the Addition of Docetaxel to Doxorubicin With Doxorubicin-Based Chemotherapy: BIG 02-98. Journal of Clinical Oncology.

[13] Dieci MV, Mathieu MC, Guarneri V, Conte P, Delaloge S, Andre F, Goubar A (2015). Prognostic and predictive value of tumor-infiltrating lymphocytes in two phase III randomized adjuvant breast cancer trials. Annals of Oncology.

[14] Ibrahim E, Al-Foheidi M, Al-Mansour M, Kazkaz G (2014). The prognostic value of tumor-infiltrating lymphocytes in triple-negative breast cancer: a meta-analysis. Breast Cancer Res Treat.

[15] Engels CC, Charehbili A, van de Velde CJH, Bastiaannet E, Sajet A, Putter H, van Vliet EA, van Vlierberghe RLP, Smit VTHB, Bartlett JMS, Seynaeve C, Liefers GJ, Kuppen PJK (2015). The prognostic and predictive value of Tregs and tumor immune subtypes in postmenopausal, hormone receptor-positive breast cancer patients treated with adjuvant endocrine therapy: a Dutch TEAM study analysis. Breast Cancer Res Treat.

[16] Reimers M, Engels C, Putter H, Morreau H, Liefers G, van de Velde C, Kuppen P (2014). Prognostic value of HLA class I, HLA-E, HLA-G and Tregs in rectal cancer: a retrospective cohort study. BMC Cancer.

[17] Krammer PH (2000). CD95's deadly mission in the immune system. Nature.

[18] The Human Protein Atlas http://www.proteinatlas.org/ENSG00000026103-FAS/tissue.

[19] Ashkenazi A, Dixit VM (1998). Death Receptors: Signaling and Modulation. Science.

[20] Waring P, Mullbacher A (1999). Cell death induced by the Fas/Fas ligand pathway and its role in pathology. Immunol Cell Biol.

[21] Reimer T, Herrnring C, Koczan D, Richter D, Gerber B, Kabelitz D, Friese K, Thiesen HJ (2000). FasL: Fas Ratio: Prognostic Factor in Breast Carcinomas. Cancer Research.

[22] Sjostrom J, Blomqvist C, NO von BK Bengtsson, Mjaaland I, Malmstrom P, Ostenstadt B, Wist E, Valvere V, Takayama S, Reed JC, Saksela E (2002). The predictive value of bcl-2, bax, bcl-xL, bag-1, fas, and fasL for chemotherapy response in advanced breast cancer. Clin Cancer Res.

[23] Mottolese M, Buglioni S, Bracalenti C, Cardarelli MA, Ciabocco L, Giannarelli D, Botti C, Natali PG, Concetti A, Venanzi FM (2000). Prognostic relevance of altered Fas (CD95)-system in human breast cancer. Int J Cancer.

[24] Debatin KM, Krammer PH Death receptors in chemotherapy and cancer. Oncogene.

[25] (2012). Comprehensive molecular portraits of human breast tumours. Nature.

[26] Lee AJ, Cunningham AP, Kuchenbaecker KB, Mavaddat N, Easton DF, Antoniou AC (2014). BOADICEA breast cancer risk prediction model: updates to cancer incidences, tumour pathology and web interface. Br J Cancer.

[27] Peter ME, Hadji A, Murmann AE, Brockway S, Putzbach W, Pattanayak A, Ceppi P (2015). The role of CD95 and CD95 ligand in cancer. Cell Death Differ.

[28] Nanda R, Chow LQM, Dees EC, Berger R, Gupta S, Geva R, Pusztai L, Pathiraja K, Aktan G, Cheng JD, Karantza V, Buisseret L (2016). Pembrolizumab in Patients With Advanced Triple-Negative Breast Cancer: Phase Ib KEYNOTE-012 Study. Journal of Clinical Oncology.

[29] Mittendorf EA, Philips AV, Meric-Bernstam F, Qiao N, Wu Y, Harrington S, Su X, Wang Y, Gonzalez-Angulo AM, Akcakanat A, Chawla A, Curran M, Hwu P (2014). PD-L1 Expression in Triple-Negative Breast Cancer. Cancer Immunology Research.

[30] Topalian SL, Drake CG, Pardoll DM (2012). Targeting the PD-1/B7-H1 (PD-L1) pathway to activate anti-tumor immunity. Current opinion in immunology.

[31] Frederick L, Page DL, Fleming ID (2002). AJCC cancer staging manual 1 edn.

[32] Bloom HJG, Richardson WW (1957). Histological Grading and Prognosis in Breast Cancer: A Study of 1409 Cases of which 359 have been Followed for 15 Years. Br J Cancer.

[33] Cerami E, Gao J, Dogrusoz U, Gross BE, Sumer SO, Aksoy BA, Jacobsen A, Byrne CJ, Heuer ML, Larsson E, Antipin Y, Reva B, Goldberg AP (2012). The cBio Cancer Genomics Portal: An Open Platform for Exploring Multidimensional Cancer Genomics Data. Cancer Discovery.

[34] Gao J, Aksoy BA, Dogrusoz U, Dresdner G, Gross B, Sumer SO, Sun Y, Jacobsen A, Sinha R, Larsson E, Cerami E, Sander C, Schultz N (2013). Integrative Analysis of Complex Cancer Genomics and Clinical Profiles Using the cBioPortal. Sci Signal.

