# Supplementary material for: Combined evaluation of the FAS cell surface death receptor and CD8+ tumor infiltrating lymphocytes as a prognostic biomarker in breast cancer
Source: Oncotarget. 2017 Jan 21;8(9):15610–20. doi: 10.18632/oncotarget.14779 (PMC5362509; doi:10.18632/oncotarget.14779)
Supplement: Supplementary file 1 [file oncotarget-08-15610-s001.pdf]

Combined evaluation of the FAS cell surface death receptor and CD8+ tumor infiltrating lymphocytes as a prognostic biomarker in breast cancer

SUPPLEMENTARY FIGURES

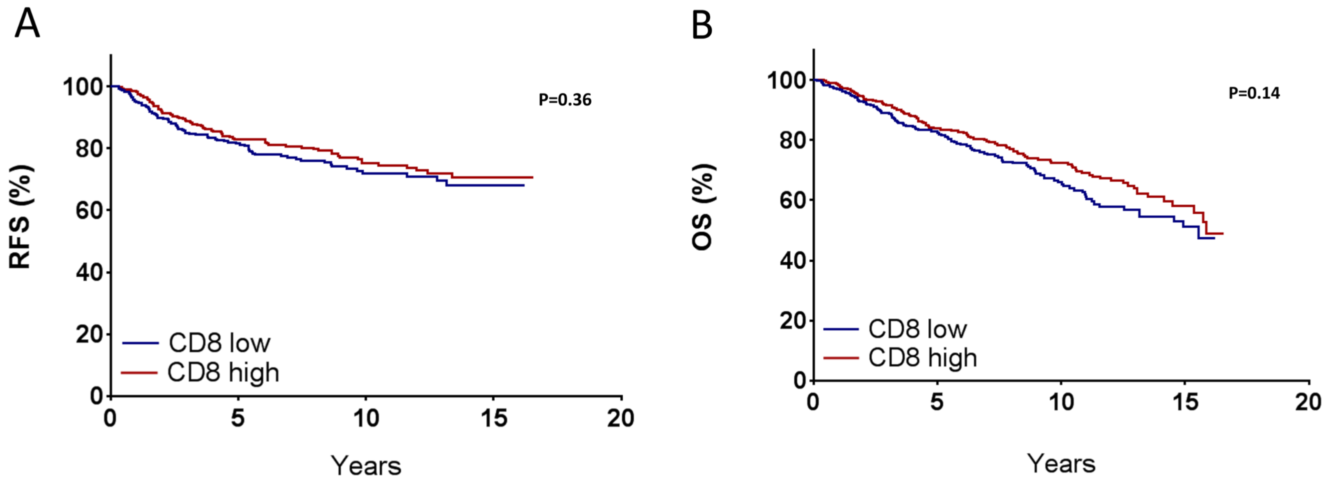

Supplementary Figure 1: Kaplan Meier survival analysis, comparing groups with either low or high presence of CD8-positive TILs in the general study population for either RFS **A.** or OS **B.** P-values represent log-rank survival test.

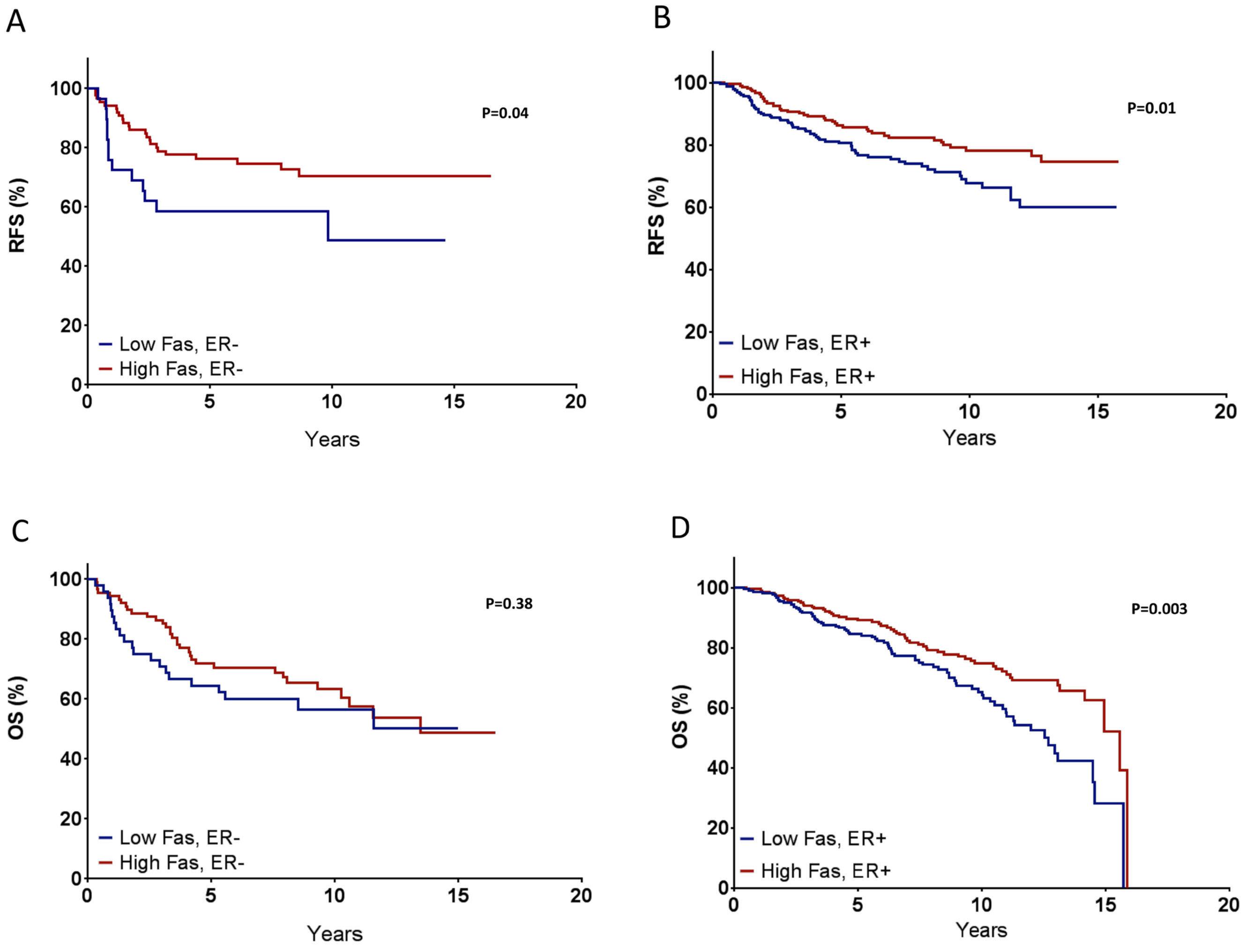

Supplementary Figure 2: The prognostic effect of FAS expression, stratified on ER-status. The effect is conserved in both ER-negative **A, C.** and ER-positive tumors **B, D.** for both RFS and OS. P-values represent log-rank survival test.

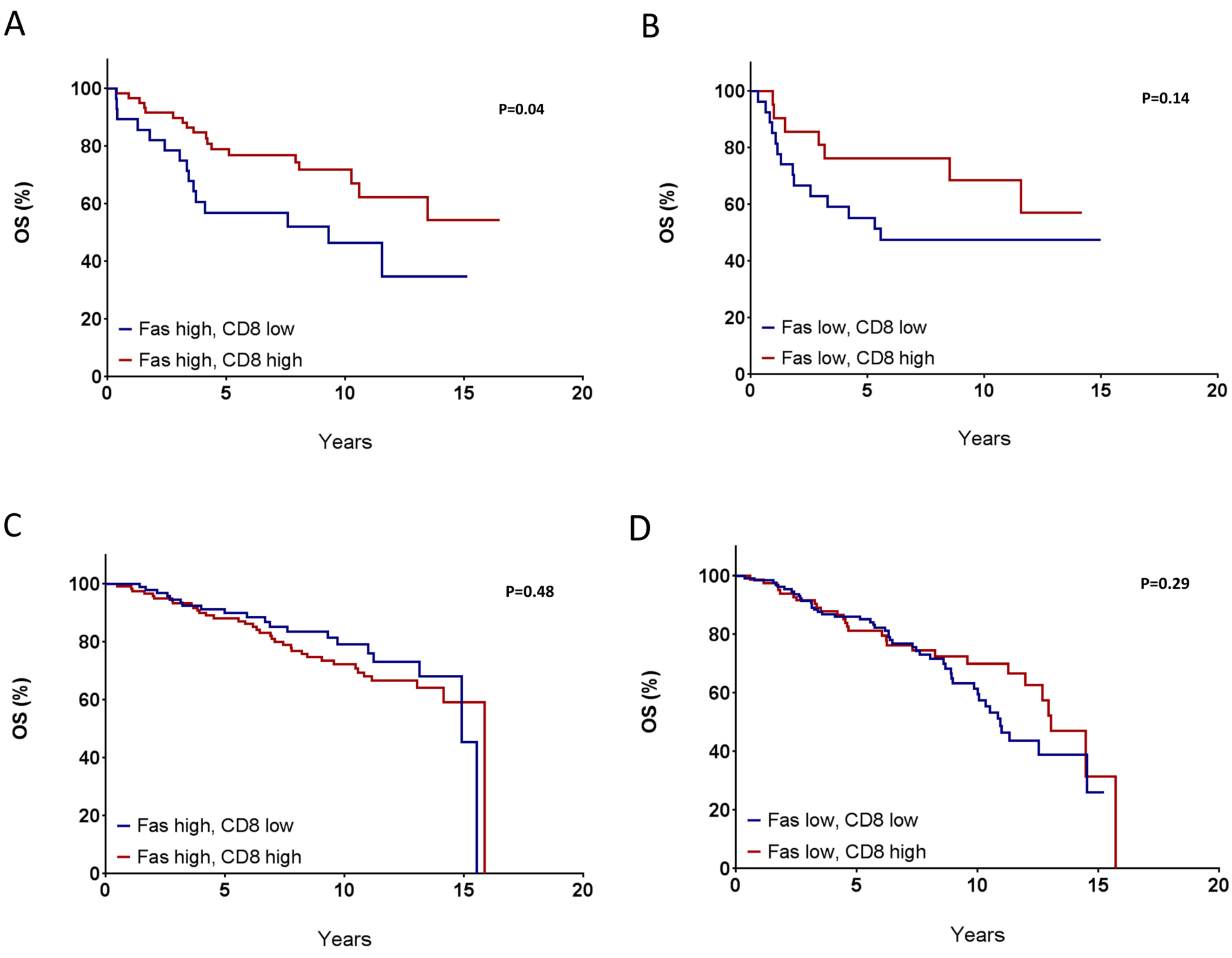

Supplementary Figure 3: The effect of CD8+ TILs on overall survival, stratified on both ER-status and FAS expression. In ER-negative tumors with high FAS expression, there is a significant benefit on OS of high CD8+ TIL presence, which is not observed in tumors with low FAS expression **B.** Furthermore, this effect is not shown in ER-positive tumors, either with high. **C.** or low **D.** FAS expression.
